# Inferring quantity and qualities of superimposed reaction rates from single molecule survival time distributions

**DOI:** 10.1038/s41598-020-58634-y

**Published:** 2020-02-04

**Authors:** Matthias Reisser, Johannes Hettich, Timo Kuhn, Achim P. Popp, Andreas Große-Berkenbusch, J. Christof M. Gebhardt

**Affiliations:** Institute of Biophysics, Ulm University, Albert-Einstein-Allee 11, 89081 Ulm, Germany

**Keywords:** Biological fluorescence, Single-molecule biophysics

## Abstract

Actions of molecular species, for example binding of transcription factors to chromatin, may comprise several superimposed reaction pathways. The number and the rate constants of such superimposed reactions can in principle be resolved by inverse Laplace transformation of the corresponding distribution of reaction lifetimes. However, current approaches to solve this transformation are challenged by photobleaching-prone fluorescence measurements of lifetime distributions. Here, we present a genuine rate identification method (GRID), which infers the quantity, rates and amplitudes of dissociation processes from fluorescence lifetime distributions using a dense grid of possible decay rates. In contrast to common multi-exponential analysis of lifetime distributions, GRID is able to distinguish between broad and narrow clusters of decay rates. We validate GRID by simulations and apply it to CDX2-chromatin interactions measured by live cell single molecule fluorescence microscopy. GRID reveals well-separated narrow decay rate clusters of CDX2, in part overlooked by multi-exponential analysis. We discuss the amplitudes of the decay rate spectrum in terms of frequency of observed events and occupation probability of reaction states. We further demonstrate that a narrow decay rate cluster is compatible with a common model of TF sliding on DNA.

## Introduction

**Figure 1:**
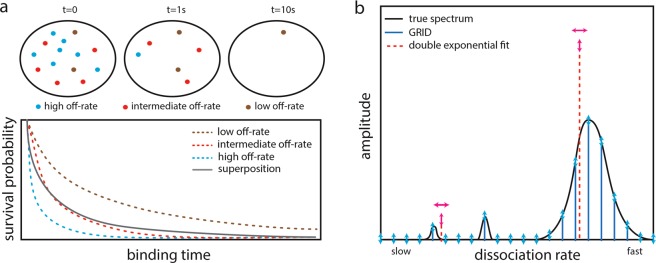
Working principle of GRID. (**a**) Sketch of a TF exhibiting three distinct dissociation processes from chromatin (upper panel). The resulting survival time distribution is a superposition of the survival times of all three processes (lower panel). (**b**) Sketch of a decay rate spectrum (black solid line) underlying a complex survival time distribution. In common multi-exponential analysis, the number of decay rates has to be guessed and their values and amplitudes are varied (red dashed lines). In contrast, GRID only varies the amplitudes of a grid of decay rates (blue solid lines). Degrees of freedom are indicated by arrows.

The actions of biomolecules are governed by thermal fluctuations and thus are intrinsically stochastic. Accordingly, interactions such as association and dissociation events of molecular species often follow Poissonian statistics with a constant probability per time, the rate constant, to occur. In this case, the experimentally accessible lifetime of the reaction is exponentially distributed. Commonly, a biomolecule engages in several different types of interaction, with each interaction type having its own reaction rate. For example, a biomolecule might bind to different protein species, to multiple sites on DNA or RNA, or to different cellular compartments. In such a scenario, not all members of a biomolecular specie will undergo the same type of interaction at any time. Rather, each biomolecule will conduct one of the multiple possible types of interaction. If the measurement determining the reaction lifetimes cannot distinguish between the different types of interaction, the resulting lifetime distribution will be multi-exponential and include reaction rates from all superimposed Poisson processes. More precisely, the lifetime distribution is a Laplace transform of the spectrum of reaction rates inherent to the biomolecule (Fig. 1a). Retrieving the underlying spectrum of reaction rates consequently evokes an inverse Laplace transformation.

The inverse Laplace transformation is an ill-posed problem for the inversion of inherently noisy, discrete distributions and numerical solutions are often unstable^1,2^. Nevertheless, algorithms treating the Laplace transform using gradient methods and appropriate regularization have been successfully developed for noisy data in NMR^3,4^ and protein folding^5^. An elegant method based on phase functions avoids fitting procedures and enables direct reconstruction of the rate spectrum of superimposed^6^ and sequential^7^ biological decay processes.

Lifetimes of biomolecular interactions are frequently measured by single-molecule fluorescence microscopy^8–25^. In such experiments, photobleaching of the fluorescent label adds a further decay path to the fluorescence signal, in addition to the dissociation processes. In single-molecule tracking, photobleaching is indistinguishable from a successful dissociation^26^. This complex kinetic scenario cannot be solved by the phase function method or current approaches of numerical inversion of the Laplace transform. Photobleaching in survival time distributions may for example be accounted for by comparison to immobile molecules such as histones H2B^11,27^, or, alternatively, the photobleaching rate constant can be directly inferred using different time-lapse conditions^9,28^.

An example of multiple superimposed reactions are transcription factor (TF) – chromatin interactions. TFs may be involved in a manifold of different binding reactions, such as binding to different specific or unspecific sequences on either free or nucleosomal DNA^29^, binding to RNA or to low-complexity domains^30^. To obtain the underlying reaction rates of TF – chromatin interactions, current analysis approaches avoid inverting the Laplace transform by describing the measured fluorescence survival time distributions with multi-exponential models with a fixed number of exponential functions but varying decay rates and amplitudes^8,10,11,31^. Such exponential fitting is robust but requires knowledge of the number of decay rates and thus is ill suited to resolve complex decay rate spectra with an unknown number of components.

Here, we tackle the problem of inverting the Laplace transform for fluorescence survival time distributions obtained by single molecule tracking subject to photobleaching. We are able to robustly infer reaction rate spectra by reducing the number of nonlinear parameters and by introducing specialized regularizations in a corresponding gradient-based optimization problem. To reduce free parameters, we apply a grid of invariable decay rates with fixed spacing but variable positive amplitudes to describe fluorescence survival time distributions. We validate our genuine rate identification (GRID) method by simulations and show that GRID enables inferring complex reaction rate spectra even if several decay rates are present. The analysis is robust for different photobleaching rates and different distributions of amplitudes. Both narrow and broad clusters of decay rates can be resolved. We apply GRID to analyse the fluorescence survival time distributions of dissociation events of the transcription factor CDX2 recorded in live cells. GRID extends the information obtained by multi-exponential fitting approaches on the number of decay rates present and the width of decay rate clusters. We discuss different interpretations of the decay rate spectrum. Moreover, using a model of TF sliding on DNA, we estimate the width of a decay rate cluster due to unbinding from multiple DNA sequences with similar binding energies. In addition, we discuss the limitations of GRID.

## Results

### Analysing superimposed reactions by GRID

We considered several parallel reactions each following Poissonian statistics with distinct dissociation rates giving rise to exponentially distributed lifetimes (Fig. 1a). We further considered measurements of reaction lifetimes by single molecule fluorescence microscopy using fluorescent labels subject to photobleaching. The corresponding survival time distribution is a superposition of exponential functions weighted by the relative occurrence of each process and enveloped by the decay of fluorescent labels (Fig. 1a, Methods, Eq. 2). Since the photobleaching rate adds to every dissociation rate, the rate spectrum obtained by an inverse Laplace transformation would be shifted by the value of the photobleaching rate, thereby impeding this approach without considering photobleaching. To correct for photobleaching, we performed time-lapse measurements, where each time-lapse condition is characterized by a sequence of short illuminations separated by a dark period of varying duration, that differently alters the photobleaching rate while leaving the dissociation rates unaltered (Methods)^9^. This measurement scheme enabled us to separate the photobleaching rate and dissociation rates in a global optimization process with an inverse Laplace transformation for each time-lapse condition.

We reduced the number of non-linear parameters in a minimization problem solving the inverse Laplace transformation by introducing a grid of densely spaced invariable decay rates with variable amplitudes (Fig. 1b and Methods). We further designed a cost function restricting the amplitudes to positive values for physical reasons. The cost function also accounted for the limited time resolution of fast dissociation rates due to the integration time of the camera, or to the criterion we used to define bound molecules, respectively. We refer to this regularization as mean decay regularization (MDR) (Methods). If the regularization is omitted, fast decay rates are used to account for noise in the first data points of the time-lapse records without compromising overall quality of the fit, since fast dissociation rates introduce negligible error at large times. The cost function can accommodate any number of time-lapse conditions in single molecule fluorescence measurements. We used the gradient method implemented in the Matlab R2017a fmincon function to solve the minimization problem corresponding to the inverse Laplace transform (Methods).

We validated our approach, GRID, using simulated survival time distributions. We simulated distributions as would be obtained by single molecule fluorescence measurements with up to 10 time-lapse conditions spanning a range of 0 s dark time up to 31.57 s dark time between two adjacent images, acquired using 50 ms exposure time and synchronous illumination. We simulated 10,000 recorded reaction events per condition (if not stated otherwise), a photobleaching rate constant *k* = *1* s^−1^, similar to experimental values for organic dyes^11,18^ and considered noise intrinsic to Poisson processes (Methods and Supplementary Table 1). To test the performance of GRID, we compared different cost functions and varied several qualities of the rate spectra including the quantity of well-separated superimposed rates, rate values and amplitudes and the width of densely spaced rate clusters.

**Figure 2:**
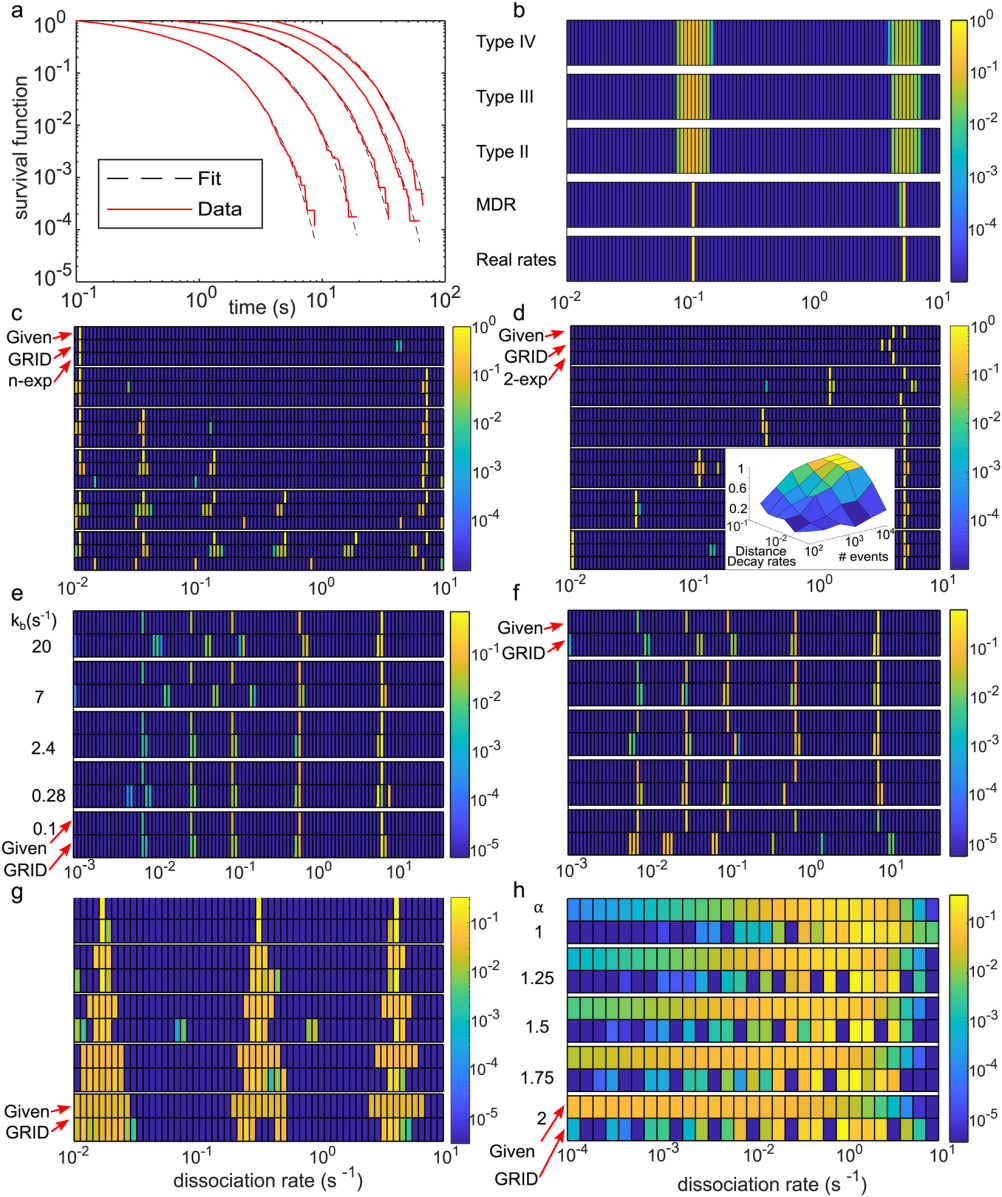
Validation of GRID by simulations. (**a**) Simulated survival time distributions with dissociation rates of 0.1 s^−1^ and 5 s^−1^ and photobleaching rate of 1 s^−1^ (red lines) and distributions obtained using the results by GRID displayed in (b) (black dashed lines). (**b**) Comparison of different regularizations (specified in Methods) used in the cost function of GRID. The amplitudes of the spectra are colour coded. (**c**–**h**) Heat maps comparing the ground truth rate spectrum (indicated as Given) used to simulate survival time distributions (1^st^ line) and the rate spectrum obtained by GRID (2^nd^ line). Where applicable we added a 3^rd^ line indicating the results of a multi-exponential approach. Simulations include a photobleaching rate of 1s^−1^, if not specified otherwise. Amplitudes are colour coded with logarithmic scale. The simulation parameters are summarized in Supplementary Table 1. (**c**) Behaviour of GRID for an increasing number of decay rates starting at *k* = 0.011 s^−1^ and separated by a factor of 4. (**d**) Behaviour of GRID for an increasing separation between two distinct decay rates with *k*_fast_ = 5 s^−1^ and *k*_slow_ varying in interval [0.01, 4] s^−1^. Inset: influence of the number of detected events and separation of decay rates on the accuracy of the inferred spectrum (Methods). (**e**) Effect of different photobleaching rate constants (indicated on the left) on inferring five irregularly spaced decay rates with different amplitudes. (**f**) Effect of varying amplitudes on inferring five irregularly spaced decay rates. (**g**) Increasing width of three decay rate clusters centred at *k*_slow_ = 0.016 s^−1^, *k*_int_ = 0.3 s^−1^ and *k*_fast_ = 3.9 s^−1^. Relative width of clusters is up to 70%. (**h**) Behaviour of GRID in the case of survival probabilities following a power-law distribution for different values of the exponent (indicated on the left).

First, we compared the performance of the MDR in our cost function with respect to generic regularizations such as the L2-norm^32^ and the L4-norm of the fitted parameters and a more specific norm that weights fitting parameters with the hyperbolic cosine (Methods). We simulated survival time distributions (1,000 events per time-lapse condition) with two superimposed reactions with rates of 0.1 s^−1^ and 5 s^−1^ (Fig. 2a and Supplementary Table 1). While all alternative regularizations showed artificial broadening of rate distributions, our MDR successfully reproduced the ground truth rate spectrum (Fig. 2a,b). We thus retained our cost function for the remainder of the study.

Second, we simulated survival time distributions (100,000 events per time-lapse condition) with an increasing number of superimposed reactions with rates between 0.01 s^−1^ and 10 s^−1^, separated by at least a factor of 4 (Supplementary Table 1). Within this range and spacing, GRID reliably identified up to six distinct reaction rates (Fig. 2c). We note that the color code in Fig. 2 is logarithmically spaced and false positive rate detections comprise less than a few percent of the total spectral mass. These false positive rates occur stochastically and might be due to noise in the simulated distributions.

Third, we investigated whether the spacing of rates influenced the performance of GRID. We simulated survival time distributions with a fast dissociation rate fixed at 5 s^−1^ and varied a slow dissociation rate between 10^−2^ s^−1^ and 4 s^−1^ (Fig. 2d and Supplementary Table 1). GRID inferred rate values reliably up to a separation by a factor of ~2, comparable to a two-exponential fit and consistent with the resolution limit of exponential analysis^33^. Analogously, we varied the fast dissociation rate between 10^−2^ s^−1^ and 10 s^−1^ while keeping the slow dissociation rate constant at 5.4·10^−3^ s^−1^ (Supplementary Fig. 1). Again, the values of both rates were accurately determined up to a separation by a factor of ~2, and comparable to a fit with a double-exponential model. To estimate the influence of the number of simulated reaction events on the accuracy of the inferred rate spectra for the case of two simulated dissociation rates with variable spacing, we quantified the overlap of ground truth spectra and GRID inferred spectra by calculating the scalar product of both in 100 independent simulations, and varied the number of simulated reaction events between 100 and 10,000 per time-lapse condition (Fig. 2d and Methods). In line with^34^, the closer the reaction rates, the more reaction events need to be observed to resolve them.

Fourth, we examined the effect of the photobleaching rate constant on the rate spectrum inferred by GRID. We simulated survival time distributions (20,000 events per time-lapse condition) with several inhomogenously spaced dissociation rates between 6·10^−3^ s^−1^ and 6 s^−1^ and increasing amplitude on the basis of experimental values (see below) (Fig. 2e and Supplementary Table 1). Below a photobleaching rate of 2.4 s^−1^, GRID fully recovered the rate spectrum. Above this value, slow dissociation rates below 0.1 s^−1^ were not accurately recovered any more.

Fifth, we examined the response of GRID to the amplitudes of reaction rates. We simulated survival time distributions with two rates of 0.035 s^−1^ and 2.44 s^−1^ and varied their amplitudes from 0% to 100% (Supplementary Fig. 1b). GRID well recovered both rates and their amplitudes. We further varied the amplitudes in our simulation on the basis of an experimental rate spectrum (see below) (Fig. 2f and Supplementary Table 1). As long as fast dissociation rates comprise more than 50% of the spectral mass, the rate spectrum can be well recovered. This is similar to previous observations using multi-exponential models^9,34^.

Sixth, we tested to which extend GRID was able to resolve rate spectra of more complex shape. Thus, we simulated survival time distributions (250,000 events per time-lapse condition) using three dense square shaped decay rate clusters at centre positions of 0.016 s^−1^, 0.3 s^−1^ and 3.9 s^−1^ and stepwise increased their width from 0% to 70% relative width (Fig. 2g). GRID recovered the width of rate clusters in most scenarios. However, a tendency to split clusters into two close sub clusters became apparent.

Since a power-law behaviour of TF – chromatin dissociation has been suggested^8,14,18^, we tested whether GRID would accurately resolve a power-law shaped ground truth. In principle, GRID is able to handle power-law distributions (Methods). We simulated several survival time distributions (100,000 events per time-lapse condition) corresponding to power-laws with exponents between 1 and 2^14,18^ including photobleaching and noise (Methods, Eq. (10)) (Fig. 2h). GRID split the broad distribution of decay rates into sub clusters. However, the resulting decay rate distribution is well distinguishable from a sparse distribution of decay rates or broad individual rate clusters (Fig. 2c-g).

### GRID analysis of CDX2 dissociation from chromatin

**Figure 3:**
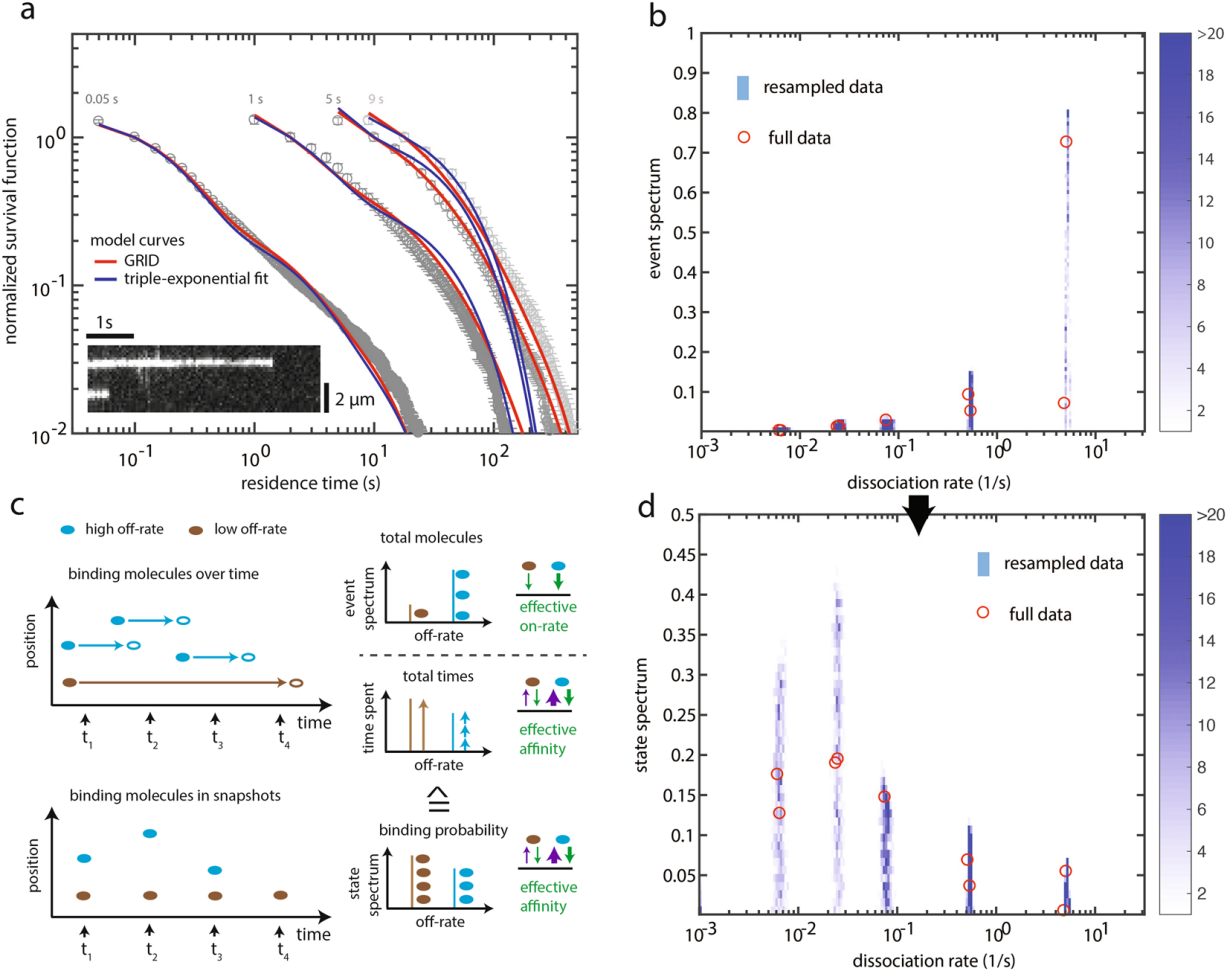
Dissociation rate spectrum of CDX2 – chromatin interactions. (**a**) Fluorescence survival time distributions of SiR-Halo-CDX2 obtained by live-cell single molecule tracking (grey symbols), fit with a tri-exponential model (blue lines) and distributions obtained using the results of GRID displayed in (**b**) (red lines). Time-lapse conditions are indicated above the distributions. The graph contains data from 10,653 molecules in 79 cells. Error bars denote s.d. (**b**) Event spectrum of CDX2 obtained by GRID using all data (red circles) and as an error estimate a heat map of 499 GRID results obtained by resampling 80% of data (blue colour code) (see Methods). (**c**) Sketch of the connection between single molecule tracking data and the corresponding event and state spectra. Upper panels: The event spectrum is obtained if molecules binding in a time interval are counted. This spectrum is a measure of an effective on-rate. To get from this kinetic rate constant to a state spectrum the binding time of these molecules has to be considered. Lower panels: The state spectrum is obtained, if molecules binding in a snapshot of time are counted. This spectrum depends on the on-rate as well as on the binding time of the molecules and is therefore a measure for the effective affinity (for details, see text and Methods). (**d**) State spectrum of CDX2 obtained by GRID using all data (red circles) and as an error estimate a heat map of 499 GRID results obtained by resampling 80% of data (blue colour code) (see Methods).

An active area of research deals with the interaction between transcription factors and chromatin. For instance, it is yet unclear how to properly distinguish and quantify the distinct modes of interaction a TF can have with the chromatin and, in particular, how many different (dissociation) rates can be resolved in live-cell experiments. Thus, after having validated our rate analysis approach with simulations, we applied GRID to survival time distributions of CDX2 dissociation from chromatin, obtained by live-cell single-molecule tracking of the fusion protein Halo-CDX2^35^ labelled with SiR-dye (Fig. 3a, Supplementary Videos 1 and 2 and Methods). We defined a Halo-CDX2 molecule as bound to chromatin if it was present within a radius of 288 nm for at least 100 ms. We recorded survival time distributions of bound molecules at four different time-lapse conditions.

GRID inferred a dissociation rate spectrum with five clearly distinct narrow dissociation rate clusters centred between 5 s^−1^ and 0.006 s^−1^ and spreading not more than two GRID units, with amplitudes strongly decreasing between ca. 80% and <5% (Fig. 3b and Supplementary Table 2). The resolution of the width of rate clusters is limited by the spacing of decay rates in GRID. To provide an estimate of the accuracy and precision of GRID we reanalysed the dataset 499 times using random 80% of the data in each time-lapse condition^36^ (Fig. 3b and Methods). This revealed a spread of decay rates within three to five GRID units. The photobleaching rate of the SiR-dye was obtained as 0.1 s^−1^. Simulated distributions using the dissociation rate spectrum extracted from the data by GRID well overlapped with the measured survival time distributions (Fig. 3a), in contrast to simulated distributions using dissociation rates obtained by fitting a tri-exponential model (Fig. 3a). Compared to common multi-exponential analysis, dissociation rates inferred by GRID better described the measurement.

To test for the influence of time-lapse conditions on the rate spectrum determined by GRID, we omitted the fastest time-lapse condition of 0.05 s in the analysis (Supplementary Fig. 2a). This time-lapse condition exclusively contains temporal information between 0.05 and 1 s. As expected, the extracted rate spectrum is devoid of the dissociation rate at 5 s^−1^, while the remaining spectrum does not change considerably (photobleaching rate was 0.4 s^−1^) (Supplementary Fig. 2b). When omitting the slowest time-lapse condition of 9 s, which contains similar temporal information than the time-lapse condition of 5 s, the extracted rate spectrum does not change considerably, as expected (photobleaching rate was 0.1 s^−1^) (Supplementary Fig. 2c,d). This analysis points towards robust inference of rate spectra by GRID.

The dissociation rate spectrum obtained by GRID yields the relative frequency with which dissociation events of a certain rate occur during an observation period (Fig. 3c). We call such a spectrum ‘event spectrum’. The amplitudes depend on the effective on-rate of the TF to the corresponding binding site and thus include information on the number of binding sites and the physical on-rate. Alternatively, information on the probability to observe a TF engaged in a certain binding state at an instantaneous time snapshot might be important. We call such a spectrum ‘state spectrum’. The event spectrum can be transformed into the state spectrum by weighting the amplitudes with the according rates (Methods). The amplitudes in the resulting state spectrum depend on the effective affinities between the TF and the corresponding binding site.

We calculated the state spectrum for Halo-CDX2 and found that TFs have a high probability to populate binding states with slow dissociation rate. While dissociation events with the transient rate of ca. 5 s^−1^ occur most frequent during an observation period (Fig. 3b), binding sites with a slow dissociation rate of ca. 0.006 s^−1^ are most often populated at any snapshot in time (Fig. 3d).

We further tested for the influence of the number of measured data points in survival time distributions on the rate spectrum determined by GRID. We successively reduced the percentage of measured data included in resampling from 80% to 20% (Supplementary Fig. 3). While the recovered decay rates spread over more GRID units for lesser data, the overall shape of the decay rate spectrum was still recovered even with only 35% of the measured data, pointing to the robustness of the method.

### Influence of TF sliding on DNA on the width of decay rate clusters

**Figure 4:**
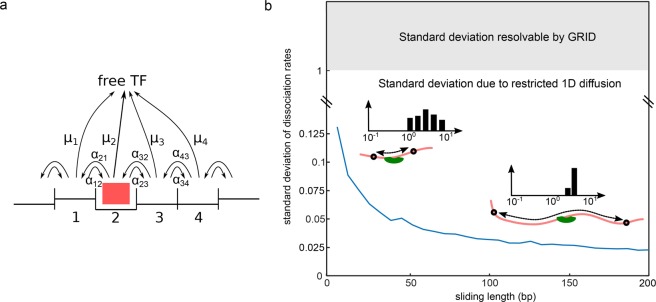
Model of TF sliding on chromatin and predicted standard deviation of dissociation rate clusters. (**a**) State diagram of a TF (red box) sliding on and dissociating from DNA. Each binding position is associated with an individual binding energy. The kinetic parameters are specified in Methods. (**b**) Standard deviation of dissociation rates as a function of sliding length. For each sliding length, dissociation rates were obtained from 500 simulations of the sliding model depicted in (**a**), where the sliding length of each segment has been kept constant but the base-pair content of each segment was varied for every simulation (for details see Methods).

It is commonly assumed that dissociation of a TF from chromatin occurs from a few specific sequences and a plethora of unspecific sequences including one or several base mismatches at various positions. To test whether GRID is sensitive to this feature of chromatin interaction, we modelled, and then simulated, the process of a TF interacting with multiple, contiguous nonspecific DNA sites (also known as 1D sliding) and compared the width of the resulting distributions to our simulations with GRID. We modelled unspecific DNA segments of variable length with dissociation from any site within the sliding segment^37–39^ (Fig. 4a and Methods). We considered a standard deviation of unspecific binding energies of 1 k_B_T compatible with sliding^40^. We found that the dissociation rate from a single segment would reduce to a single value if fast 1D diffusion took place. When considering several separate segments of equal length but different base sequence, the corresponding dissociation rates combined to a narrow cluster, due to stochasticity in the base pair content of different segments. The width of this decay rate cluster anti-correlated with the length of the segments (Fig. 4b). This is due to averaging of individual dissociation rates on the DNA segment. Even small sliding segments resulted in cluster widths well below the resolution of GRID given by the spacing of invariable decay rates. Our calculations suggest that unspecific TF – DNA interactions in the presence of sliding result in a narrow decay rate cluster currently not resolvable by GRID.

## Discussion

### GRID reveals rate spectra underlying complex survival time distributions

We introduced GRID, an approach to extract reaction rates from experimentally measured fluorescence survival time distributions of complex superimposed reactions. GRID robustly identifies the number and amplitudes of reaction rates and gives information on the width of rate clusters, even if lifetime measurements are aggravated by photobleaching of fluorescent labels. Such distorting additional photobleaching rates hamper the use of previously reported approaches to tackle the inverse Laplace transformation of survival time distributions^3–6^ We note that, while we validated and applied GRID to data sets including photobleaching and several time-lapse conditions, it should in principle also be applicable to individual survival time distributions already corrected for photobleaching.

GRID has the advantage that the number of decay rates in the biological system does not have to be guessed. This is a major drawback of current multi-exponential analysis schemes using a small number of decay rates^11,31,41^. Our simulations suggest that GRID, despite being the more complex approach, does not come with a loss in accuracy in a situation where the number of decay rates is known. In contrast, if more than three decay rates are present, GRID rather outperforms multi-exponential analysis schemes. We found that GRID well resolved up to six distinct decay rates in a range from 10 s^−1^ to 10^−3^ s^−1^. A second advantage of GRID is that it can reveal the width of reaction rate clusters, information intrinsically inaccessible to multi-exponential analysis schemes using a small number of decay rates.

GRID is currently restricted to superimposed reactions following Poissonian statistics with positive amplitudes. Thus, GRID is not applicable to arbitrary survival time distributions (Methods). Due to computation costs, the number of rates in the grid is currently limited to 200 (Methods). Consequently, the resolution to identify decay rates is limited, and oftentimes GRID splits a single decay rate onto two adjacent grid positions. Compared to Zhou *et al*.^6^, GRID converts the inverse Laplace transformation into an optimization problem, with the accompanying disadvantage of a large number of degrees of freedom. This required introducing a robust regularization. Additionally, a large number of measurements are advisable. In simulations including two decay rates, 5,000 data points in each time-lapse condition allow very accurate recovery of rates. Our analysis of experimental data suggests that overall 10,000 data points are sufficient to robustly infer five decay rates. While GRID well allows distinguishing narrow decay rate clusters from broad clusters or a power-law distribution, it is limited in identifying the shape of broad clusters or distributions with high accuracy. We summarized the resolution limits of GRID in Table 1.

**Table 1:** Estimated resolution limits of GRID for several parameters. The values for GRID are estimated from Fig. 2. The values for the multi- exponential approach are estimated from Fig. 2c,d and^34^.

parameter	maximum rate	minimum rate	minimum distance between two rates	accuracy of amplitude	accuracy of rate
GRID	<10/s @ 20fps	>0.001/s for live cell	>4 fold	ca. 10%^a^	ca. 10%
multi-exponential	<10/s @ 20fps	>0.001/s for live cell	>4 fold	ca. 10%^a^	ca. 5% for two rates
**parameter**	**photobleaching rate**	**events in fastest rate**	**width of rate cluster**	**number of rates**	
GRID	≤2.4/s	>50%	✓	≤6	
multi-exponential	n.d.	>50%	not possible	≤3	

^a^relative to the other amplitudes.

### Rates of CDX2 – chromatin dissociation

For the dissociation of CDX2 from chromatin, GRID resolved five narrow dissociation rate clusters corresponding to chromatin residence times between 0.2 s and 170 s. All five interaction times appear necessary for a full description of the measured survival time distributions, as multi-exponential fitting using three dissociation rates as reported previously for different TFs^31,41^ failed to fully recover the measured survival time distributions.

The amplitudes in the event spectrum of CDX2 are given by the effective on-rates of complex formation of CDX2 with corresponding binding sites on chromatin. If an identical kinetic on-rate is assumed for all binding sites, the event spectrum has its origin in the relative abundance of binding sites giving rise to a certain dissociation rate. Under this assumption, in the case of CDX2, ca. 80% of accessible binding sites would exhibit the shortest measured off-rate. In contrast, the amplitudes in the state spectrum are given by the effective affinities of CDX2 to corresponding binding sites on chromatin. The state spectrum reveals that on average only approximately 6% of all bound molecules are engaged in such short interactions while ca. 70% are engaged in the two interactions of longest duration.

A fast rate above 1 s^−1^ of TF – chromatin interactions has previously been identified as binding of the TF to unspecific DNA sequences^14,20,42^. Although we do not have experimental evidence, by analogy, CDX2, too, might exhibit transient unspecific and stable specific binding to chromatin. However, we cannot exclude that also slow rate constants include unspecific dissociation processes. Due to the global accuracy of GRID, it might become possible to uniquely assign certain molecular interactions to certain dissociation rates in future studies.

For unspecific LacI and TetR – chromatin interactions, a power law was used to describe a large section of the survival time distribution^14,18^. Within this time section, the rate spectrum will be a continuous distribution, potentially representing a multitude of co-occurring different dissociation rates. For CDX2, despite the capability of GRID to hint at broad clusters, we did not observe continuous rate distributions but rather well-separated narrow dissociation rate clusters. These different observations probably reflect TF-specific kinetic behaviours.

The model we present for TF sliding on DNA might serve as an example for a system in which numerous different dissociation processes do not lead to a broad dissociation rate spectrum but a rate cluster with narrow width due to quasi-averaging. In fact, the width of this cluster would be smaller than one GRID unit.

## Materials and Methods

### Model for the survival time function of an ensemble of chromatin-bound fluorescently labelled TFs

We assume that dissociation of a TF from any bound state, in particular from a bound DNA sequence, follows Poissonian statistics with a dissociation rate constant *µ*_*l*_ characteristic for this particular state. We further assume that the TF may bind to a multitude of different DNA sequences, both unspecific and specific. The probability of a particular dissociation event to occur be *S*_*l*_.

For independent dissociation processes, the resulting survival time function of an ensemble of TFs is a superposition of individual dissociation processes

1 $$N(t)={N}_{0}\mathop{\sum }\limits_{l=1}^{L}{S}_{l}\exp (\,-{\mu }_{l}t)$$

for the remaining bound population *N* at time *t* if *N*_0_ TFs were bound at time *t* = 0. *N*_0_∙*S*_l_ is the number of TFs in the ensemble that exhibits the dissociation rate constant *µ*_l_. The total number of dissociation processes is denoted by *L*.

So far, we assumed that the survival time function of bound TFs is only determined by dissociation. However, in single molecule fluorescence experiments, the TF is identified by a fluorescent label prone to photobleaching. Thus, the experimentally observed termination of a bound state may be due to photobleaching of the fluorescent label or dissociation of the TF. We assume that photobleaching also follows Poissonian statistics.

The fluorescence survival time function observed in experiments then reads

2 $$N(t)={N}_{0}\exp (\,-\,kt)\mathop{\sum }\limits_{l=1}^{L}{S}_{l}\exp (\,-{\mu }_{l}t)$$

where *k* is the photobleaching rate constant. According to this equation, only the sum *k* + *μ*_*l*_ can be inferred from the fluorescence survival time distribution. To separate photobleaching from dissociation, we performed time-lapse measurements^9^. There, by introducing varying dark times between two images, the relative contributions of illumination time-dependent photobleaching and real time-dependent dissociation can be separated.

### Simulation of TF dissociation kinetics

We simulated survival time distributions of TFs with effective dissociation rate constants ${k}_{{\rm{eff}},{\rm{l}}}=a/{\tau }_{tl}+{k}_{{\rm{off}},{\rm{l}}}$ accounting for dissociation with dissociation rate constant ${k}_{{\rm{off}},{\rm{l}}}$ and with the photobleaching number $a=k\,{\tau }_{int}$, the camera integration time ${\tau }_{int}\,$and the time-lapse period ${\tau }_{tl}$. Different ${k}_{{\rm{off}},{\rm{l}}}$ occurred with probability ${S}_{l}$. We first generated a random number with uniform distribution to draw the ${k}_{eff,l}$ from the probability distribution $S$. Next, we generated a new random number from an exponential distribution with the constant ${k}_{eff,l}$ to obtain the time at which the TF dissociated. This time entered a distribution with a bin-size corresponding to the time-lapse period. We repeated this procedure N times to obtain a survival time distribution of *N* TFs. To obtain a complete dataset, we repeated this procedure for various time-lapse periods ${\tau }_{tl}$. Simulations were conducted in MATLAB R2017a.

### A Method for the inverse Laplace transform

To determine the dissociation rate spectrum of the TF, we could in principle fit the fluorescence survival time function including photobleaching, Eq. 2, to the measured distributions obtained from several time-lapse conditions. However, we neither know the number of dissociation processes $L$ nor can we ensure numerical stability of a fit with multiple degrees of freedom that lead to nonlinear gradients. To ensure unbiased and robust inference of dissociation rates in Eq. 2, we reduced the number of free parameters by applying a grid of *I* invariable dissociation rates with fixed spacing and numerically determined the probabilities *S*_*i*_ of each dissociation rate. Summing up, the number of unknown parameters is $I+1$, namely [k, S]. Since *I* is usually larger than the number of observables in time-lapse measurements, the fitting problem is underdetermined. Thus, to obtain a unique solution, we applied regularizations based on basic physical considerations and time resolution constraints of the measurement process.

As first regularization, we introduced the constraint *S* ≥ 0 of non-negative probabilities and *k *≥ 0 of a non-negative photobleaching rate constant. This ensures our model is monotonically decreasing, as expected from superimposed Poisson processes. As second regularization, we accounted for the integration time *τ*_int_ of the camera used to record fluorescent light, or to the criterion we used to define bound molecules, respectively. These times limit the time resolution of fast dissociation rates (*µ*_*i*_ > *τ*_int_^−1^). As a mathematical measure of this limitation, we introduced the time dependent expectation value of the dissociation rate < *µ* > of the bound TF population

3 $$\langle \mu \rangle (t)=\frac{{\sum }_{i}{\mu }_{i}{S}_{i}\exp (\,-{\mu }_{i}t)}{{\sum }_{i}{S}_{i}\exp (\,-{\mu }_{i}t)}$$

where the value ${S}_{i}{{\rm{e}}}^{-{\mu }_{i}t}/\sum _{i}{S}_{i}{{\rm{e}}}^{-{\mu }_{i}t}$ may be interpreted as the time dependent probability to find a TF that exhibits the dissociation rate *µ*_*i*_ at time *t*. We then introduced the expression

4 $$\langle \mu \rangle ({\tau }_{\mathrm{int}})-\langle \mu \rangle (2{\tau }_{\mathrm{int}})$$

to describe the change of the mean dissociation rate in the dead time of our measurement. By minimizing this quantity, we reduced the number of degrees of freedom during our dead time and thereby avoided overfitting. We refer to this regularization as the mean decay regularization (MDR).

We next defined the difference between the fluorescence survival time function and the measured distribution of the m-th point in the n-th time-lapse record, *∆f*_*nm*,_ as

5 $$\varDelta {f}_{nm}=\frac{{f}_{nm}^{{\rm{Measurement}}}}{{f}_{n2}^{{\rm{Measurement}}}}-\frac{{f}_{nm}^{{\rm{Fit}}}}{{f}_{n2}^{{\rm{Fit}}}}$$

where we normalized the values of the fitted and measured distributions to the population at the second time point of a time-lapse record to eliminate the unknown amount of the initial population.

Our model function ${f}_{nm}^{{\rm{Fit}}}$ is given by the superposition of $I$ exponential functions

6 $${f}_{nm}^{{\rm{Fit}}}={N}_{0,n}\exp (\,-\,a\cdot m)\mathop{\sum }\limits_{i=1}^{I}{S}_{i}\exp (\,-{\mu }_{i}\cdot m\cdot {\tau }_{tl,n})$$

where ${\tau }_{tl,n}$ is the duration of the n-th time-lapse.

We further introduced the cost function *L* of the fitting problem, which consists of the difference between measurement and theoretical function, *∆f*_*nm*_, and the regularization of the mean dissociation rate

7 $$L={\sum _{n,m}\Delta {f}_{nm}^{2}+H\times [\langle \mu \rangle ({\tau }_{\mathrm{int}})-\langle \mu \rangle (2{\tau }_{\mathrm{int}})]}^{2}$$

Since both *∆f*_*nm*_ and the regularization contribute to the same cost function, we introduced the empirical parameter *H* to limit the influence of the regularization.

The complete optimization problem finally is

8 $$\min (L)\,{\rm{where}}\,{\rm{S}}\ge 0,{\rm{k}}\ge 0$$

We solved this optimization problem with the gradient-based method fmincon solver with the sequential quadratic programming algorithm of the Matlab R2017a optimization toolbox, to find the spectrum of dissociation rates of TF-chromatin dissociation. Typically, to solve the optimization problem, the gradient of the cost function is estimated numerically, which here would result in a computation time of minutes on a standard computer. We decreased this time ca. ten-fold to several seconds by providing an analytical expression for the gradient. For resampling, the time demand increases according to the number of resampling-runs performed.

Alternatively, we tested the cost functions of the L2-norm (Type II) $\sum _{n,m}\Delta {f}_{nm}^{2}+\sum _{i}{S}_{i}^{2}$, the L4-norm (Type III) $\sum _{n,m}\Delta {f}_{nm}^{2}+\,\sum _{i}{S}_{i}^{4}$ and a more specific norm that weights the fitting parameters with the hyperbolic cosine $\sum _{n,m}\Delta {f}_{nm}^{2}+\,\sum _{i}{S}_{i}\,\cosh (0.1\cdot {k}_{i})$ (Type IV).

### Application of GRID to power-law functions

In GRID, we restricted ourselves to positive dissociation rates, positive amplitudes and a positive photobleaching rate. Therefore, GRID can be applied to a certain type of model-functions. The model functions as well as the absolute value of their derivatives have to decay strictly monotonously. In particular, we show here that GRID can be applied to power-law models.

We construct a survival function by calculating the power-law

9 $$f(t)={(1+\frac{{k}_{0}t}{\alpha })}^{-\alpha }$$

where ${k}_{0}$ is a constant that shifts the pole to $t < 0$. The number $\alpha$ needs to be larger than one so that the average binding time of the TF converges. This model converges to a single exponential function in the limit $\alpha \to \infty$. We analytically calculated the spectrum *S(k)* of Eq. (9) as

10 $${(1+\frac{{k}_{0}t}{\alpha })}^{-\alpha }={\int }_{0}^{\infty }S(k)\cdot \exp (\,-\,kt){\rm{d}}k=\Gamma {(\alpha )}^{-1}{(\tfrac{\alpha }{{k}_{0}})}^{\alpha }{\int }_{0}^{\infty }{k}^{\alpha -1}\exp (\,-\,\alpha k/{k}_{0})\cdot \exp (\,-\,kt)\,{\rm{d}}k$$

To check whether the time-lapse approach combined with GRID can recover such a power-law we calculated a survival time distribution according to

11 $${f}_{bleach}(t)=\exp (\,-\,{k}_{b}{\tau }_{\mathrm{int}}/{\tau }_{tl}t)\cdot {(1+\frac{{k}_{0}t}{\alpha })}^{-\alpha }$$

To introduce noise we stochastically resampled this survival function.

### Calculation of the state spectrum

The event spectrum yields the relative frequency ${S}_{i}$ of events exhibiting the dissociation rate ${k}_{off,i}$. To calculate the state spectrum, we considered the frequency of measured events originating from a certain binding site with dissociation rate ${k}_{off,i}$ and corresponding on-rate ${k}_{on,i}^{eff}$. We assumed that the number of observed events is proportional to this effective association rate which yields

12 $${S}_{i}\propto {k}_{on,i}^{eff}$$

For a number of unoccupied binding sites ${D}_{i}\,$, the relative frequency scales with this number

13 $${S}_{i}\propto {D}_{i}{k}_{on,i}^{eff}$$

Division by the respective dissociation rate yields the effective affinity ${K}_{eff,i}$ which comprises the binding affinity of the TF and the number of free TFs and unoccupied binding sites.

14 $$\frac{{S}_{i}}{{k}_{off,i}}\propto \frac{{D}_{i}{k}_{on,i}^{eff}}{{k}_{off,i}}={K}_{eff,i}$$

To obtain the amplitudes ${{S}_{i}}^{\ast }$ of the state spectrum, Eq. (14) has to be normalized. We find:

15 $${{S}_{i}}^{\ast }=\frac{\tfrac{{S}_{i}}{{k}_{off,i}}}{{\sum }_{m=1}^{M}\tfrac{{S}_{m}}{{k}_{off,m}}}=\frac{{K}_{eff,i}}{{\sum }_{m=1}^{M}{K}_{eff,m}}=\frac{{N}_{bound,i}}{{\sum }_{m=1}^{M}{N}_{bound,m}}$$

Comparing the effective affinities yields the normalized number ${N}_{bound,i}$ of molecules bound to binding sites with the dissociation rate ${k}_{off,i}$.

### Model of TF sliding on DNA

In our model of TF-DNA dissociation, we assumed that the TF binds to a free segment of DNA with a length of *N* base pairs restricted by roadblocks at the edges of the segment^43,44^. Within the DNA segment, the TF may assume *N* different binding positions (Fig. 4). The TF slides between binding positions within this segment by 1D diffusion. The TF can leave the segment by dissociating from any position within the DNA segment. We further considered the variance *σ*^2^ of DNA binding energies in units of *k*_*b*_*T*. This variance in binding energies leads to dissociation rates that are normally distributed around the mean dissociation rate $\mu$ with a standard deviation $\sigma$. The variance of unspecific binding energies was previously estimated to be *σ* < = 1 *k*_*b*_*T*^40^. We ascribed a random dissociation rate from this distribution to each TF position within the DNA segment.

The rate of sliding of the TF from state (or position) *i* to *j* be α_ij_. The ratio of α_ij_ and α_ji_ is determined by the energy difference between the two positions, which in turn is determined by the dissociation rates of the TF from DNA at positions i and j. To find values for α_ij_ and α_ji_, we assumed that the transition rate to a lower binding energy level is given by the sliding rate, while the transition to a higher binding energy level is limited by the energetic gap between the two levels. With this assumption we calculated the transition rates according to the law of detailed balance

16 $$\begin{array}{c}{\alpha }_{i,i+1}=\{\begin{array}{cc}\beta \cdot {\mu }_{i}/{\mu }_{i+1} & {\mu }_{i} < {\mu }_{i+1}\\ \beta  & {\mu }_{i} > {\mu }_{i+1}\end{array}\\ {\alpha }_{i+1,i}=\{\begin{array}{cc}\beta \cdot {\mu }_{i}/{\mu }_{i+1} & {\mu }_{i} > {\mu }_{i+1}\\ \beta  & {\mu }_{i} < {\mu }_{i+1}\end{array}\\ {\alpha }_{i,j}=0\,{\rm{f}}{\rm{o}}{\rm{r}}\,|i-j|\ne 1\end{array}$$

where *β* is a mean sliding rate. As described in^45^, the Kolmogorov formalism may be used to model the dynamics of the TF on DNA. We found the time-dependent probability *p*_*i*_ of the TF to be in state *i*

17 $${\dot{p}}_{i}=-(\mu +{\alpha }_{i,i+1}+{\alpha }_{i,i-1}){p}_{i}+{\alpha }_{i-1,i}{p}_{i-1}+{\alpha }_{i+1,i}{p}_{i+1}$$

The observable dissociation rates are determined by the eigenvalues of the eigenvalue-problem and their amplitudes are determined by the solution of the time dependent probability. We calculated these amplitudes by introducing the initial condition $\overrightarrow{p}(0)={\overrightarrow{e}}_{n}$, which is the unitary vector in *n*-th direction. This initial condition states the initial position of the TF after association to the DNA segment. In this model of TF sliding, the amplitudes of all except one eigenvalue vanish. Thus, from a single DNA segment, we obtained only one effective dissociation rate.

Measurements in bacteria and *in vitro* found a diffusion constant of 1D sliding on the order of 0.01 μm^2^s^−1^ ^38,43,44^. Based on our theoretical modelling^45^, this results in a mean sliding rate *β* = 10^+4^s^−1^, which indicates the rate at which the TF transits to the next base-pair without detaching from DNA. The sliding length was previously estimated to be on the order of 45 base pairs in bacteria^43^.

To describe overall TF binding in the nucleus, we considered 500 independent unspecific DNA segments of equal length but different base pair content. As above, each segment contributed a single dissociation rate corresponding to the particular dissociation rate distribution of this segment of DNA. Due to the stochastic base pair composition drawn for each segment, the mean dissociation rates of different segments form a narrow cluster of dissociation rates.

### Quantitative comparison between rate spectra

To quantify the resemblance between inferred spectrum and ground truth in Fig. 2d and Supplementary Fig. 1, we calculated the scalar product of these two spectra. This value is high if the rates are at the same position and low if the rates are shifted with respect to each other. The scalar product in principle is zero if two rates are shifted by one increment in GRID. We relaxed this fact since a single rate is oftentimes split up into two neighbouring rates in our simulations due to the limited resolution of GRID. We therefore allowed a shift of up to 3 GRID units and assigned a value equal to the one obtained if no shift was present.

We calculated the scalar product for 100 stochastic simulations with identical parameters to obtain the fraction of inferred results with a matching spectrum, where we defined a matching spectrum to have a scalar product larger than 0.5. The resulting values are represented in the insets of Fig. 2d and Supplementary Fig. 1 as a function of the number of simulated events and of the separation of decay rates.

### Cell culture and preparation

NIH3T3 cells were cultured and prepared as described in^35^. Cells with stable integration of Halo-CDX2 under doxycycline-induced expression control (kindly provided by David Suter, EFPL, Lausanne, Switzerland) were seeded one day before experiments on a closable Delta-T glass bottom dish to prevent evaporation (Bioptechs, Pennsylvania, USA). Expression of Halo-CDX2 was induced by adding 10 ng/ml doxycyclin to the medium four hours before imaging. Cells were stained with SiR-dye (kindly provided by Kai Johnson, EFPL, Lausanne, Switzerland) with a final concentration of 3 pM shortly before imaging according to the Halo-tag protocol (Promega). We tested for specificity of the SiR-Halo-tag dye and did not observe any SiR-Halo-dye signal in the cell nucleus in an NIH3T3 cell line not carrying the Halo-tag fusion protein insert (Supplementary Fig. 4).

### Live cell single molecule imaging and tracking

Single molecule fluorescence imaging was performed as described previously^46^. In brief, light of a 638 nm laser (IBEAM-SMART-640-S, 150 mW, Toptica, Gräfelfing, Germany) was used to set up a highly inclined illumination pattern on a conventional fluorescence microscope (TiE, Nikon, Tokyo, Japan) using a high-NA objective (100×, NA 1.45,Nikon, Tokyo, Japan). We calculated the intensity to be approximately 1.5 kW/cm². Emission light had to pass a multiband emission filter (F72–866, AHF, Tübingen, Germany) and was subsequently detected by an EMCCD camera (iXon Ultra DU 897U, Andor, Belfast, UK) with 50 ms integration time. For time-lapse imaging, dark-times were controlled by an AOTF (AOTFnC-400.650-TN, AA Optoelectronics, Orsay, France). Temperature control was realized by the Delta-T system (Bioptechs, Pennsylvania, USA) and an additional objective collar (Thermo Technologies, Rohrbach, Germany).

Cells were prepared for imaging as detailed above and kept in OptiMEM medium at 37° during imaging for up to two hours of measurement time per dish. In each cell on average 482 molecules were detected during an average imaging period of 8 minutes. Single molecule spot detection and tracking was performed as described in^46^. In brief, we detected potential single molecules based on their fluorescence intensity compared to background fluorescence. Localization was performed using a 2D Gaussian fit. Halo-TF molecules were identified as bound molecules if they did not leave a radius of 288 nm for 3 (50 ms time-lapse) or 2 (other time lapse conditions) consecutive frames. Fluorescence survival time distributions were compiled from these tracking data.

### Resampling

To estimate accuracy and precision of a GRID result, we analysed a set of 80% randomly chosen values from the measured survival time distributions and repeated this process 499 times^36^. We plotted the resulting GRID spectra as a heat map that shows how often a certain spectral value was obtained in the 499 repetitions. If a spectral value was obtained less than two times, we omitted it.

## Supplementary information

Supplementary Video 1.

Supplementary Video 2.

Supplementary Information.

## Data Availability

Data supporting the findings of this manuscript will be available from the corresponding author after publication upon reasonable request. All raw single particle tracking data are freely available in Matlab and csv file format at 10.5061/dryad.19st68k.

## Code availability

The GRID software is freely available. A MatLab version of GRID and GRID simulation packages are available at https://gitlab.com/GebhardtLab/GRID.
