# Supplementary material for: Inferring quantity and qualities of superimposed reaction rates from single molecule survival time distributions
Source: Sci Rep. 2020 Feb 4;10:1758. doi: 10.1038/s41598-020-58634-y (PMC7000831; doi:10.1038/s41598-020-58634-y)
Supplement: Supplementary file 3 — Supplementary Information. [file 41598_2020_58634_MOESM3_ESM.pdf]

Supplementary Information

Inferring quantity and qualities of superimposed reaction rates from single molecule survival time distributions

Matthias Reisser*, Johannes Hettich*, Timo Kuhn, Achim P. Popp, Andreas Große-Berkenbusch and J. Christof M. Gebhardt[#]
Institute of Biophysics, Ulm University, Albert-Einstein-Allee 11, 89081 Ulm
*These authors contributed equally to the work
[#]To whom correspondence should be addressed: christof.gebhardt@uni-ulm.de

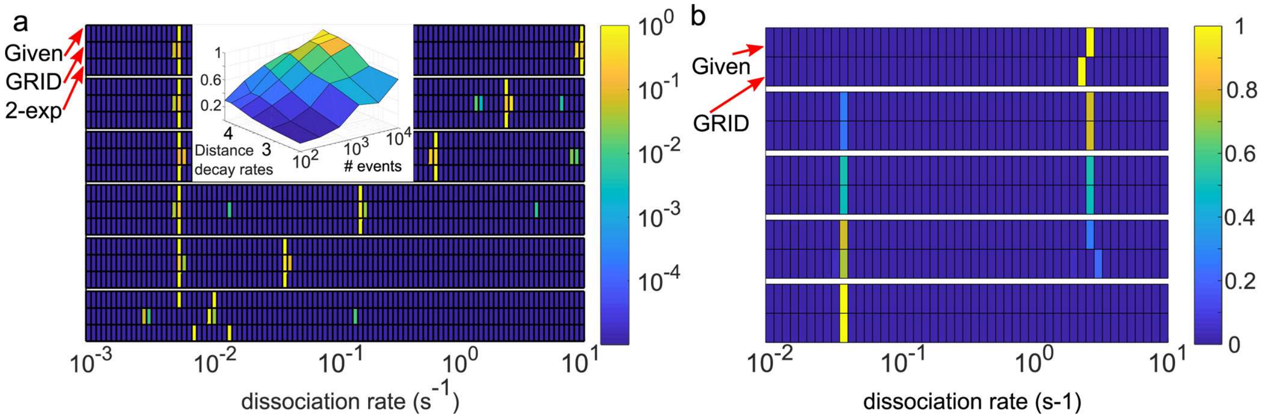

Supplementary Figure 1: Inference of positions and amplitudes of two distinct decay rates. (a-b) Heat maps comparing the ground truth (indicated by "Given") rate spectrum used to simulate survival time distributions and the rate spectrum obtained by GRID or a bi-exponential decay model. Amplitudes are colour coded with logarithmic scale. Simulations include a photobleaching rate of $1 s^{-1}$. Simulation parameters are summarised in Supplementary Table 1. (**a**) Variable separation between two distinct decay rates $k_{slow} = 5.4 \cdot 10^{-3}$ s^{-1} and k_{fast} in the interval $[10^{-2},10]$ s^{-1}. Inset: influence of the number of detected events and separation of decay rates on the accuracy of the inferred spectrum (details in Methods). **(b)** Two distinct decay rates ($k_{slow} = 0.035$ s^{-1} and $k_{fast} = 2.44$ s^{-1}) with variable amplitudes. Since rates identified by GRID oftentimes split between two positions within the applied grid of dissociation rates (GRID units) including and next to the ground truth due to limited resolution, we locally integrated over three adjacent GRID units in the spectrum to compare amplitudes.

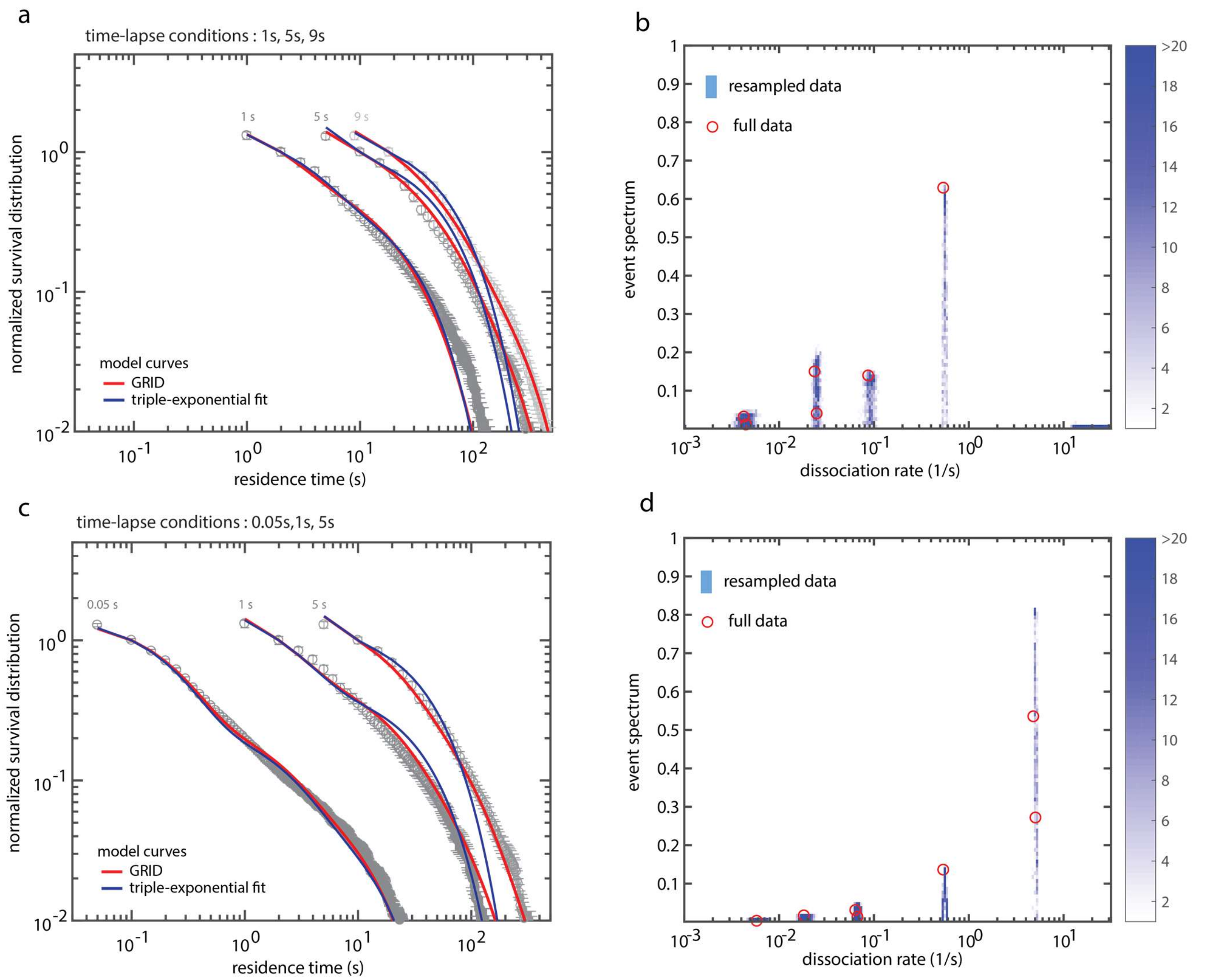

Supplementary Figure 2: Influence of time-lapse conditions on GRID rate spectra. (a,c) Fluorescence survival time distributions of SiR-Halo-CDX2 obtained by live-cell single molecule tracking (grey symbols), fit with a tri-exponential model (blue lines) and distributions obtained using the results by GRID displayed in (b,d) (red lines). Compared to the full data set in Figure 3, some time-lapse conditions (indicated above the distributions) were left out in the analysis to evaluate their individual information content. **(a)** The fastest time-lapse condition was omitted. The graph contains data from 4,022 molecules in 50 cells. Error bars denote s.d. **(b)** Event spectrum of CDX2 obtained by GRID using data from (a) (red circles) and as an error estimate a heat map of 499 GRID results obtained by resampling 80% of data (blue colour code) (see Methods). Compared to the analysis of the full data set in Figure 3, the fastest rate cluster disappeared. Renormalization of the remaining spectrum led to changes in the spectral weight of the remaining clusters. **(c)** The slowest time-lapse condition was omitted. The graph contains data from 9,449 molecules in 66 cells. Error bars denote s.d. **(d)** Event spectrum of CDX2 obtained by GRID as in (b) using data from (c). Compared to Figure 3, omitting a time-lapse conditions with redundant temporal information did not significantly change the rate spectrum.

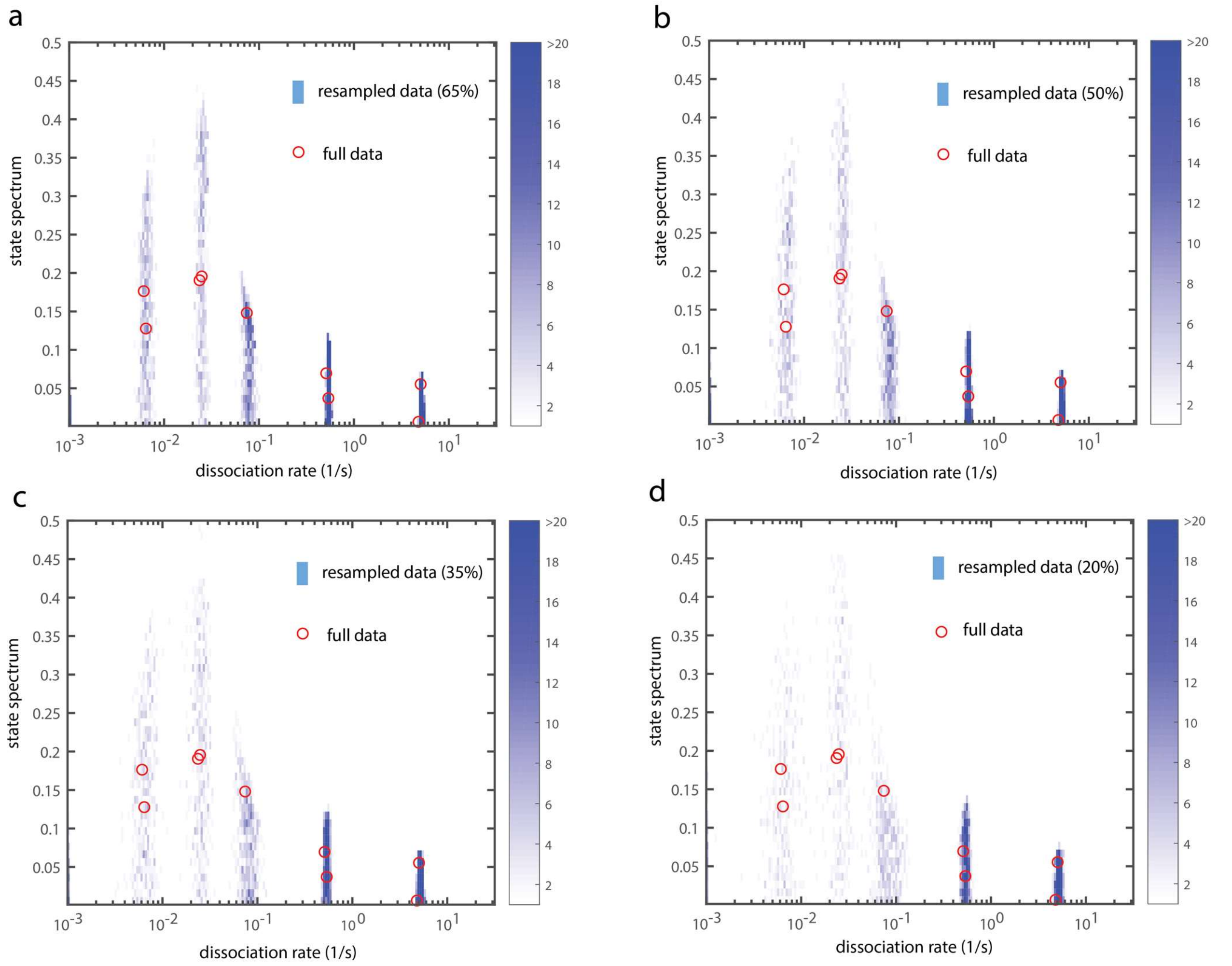

Supplementary Figure 3: Influence of the number of measured data points on GRID rate spectra. State spectra of CDX2 obtained by GRID using all data from Figure 3a (red circles) and as an error estimate a heat map of 499 GRID results obtained by resampling (**a**) 65%, (**b**) 50%, (**c**) 35% and (**d**) 20% of data (blue colour code) (see Methods). When resampled with less than 35% of data, the spectrum shows broadening and weak overlapping of dissociation rate clusters. This indicates that the number of data points in this case is not sufficient any more to calculate a conclusive state – spectrum.

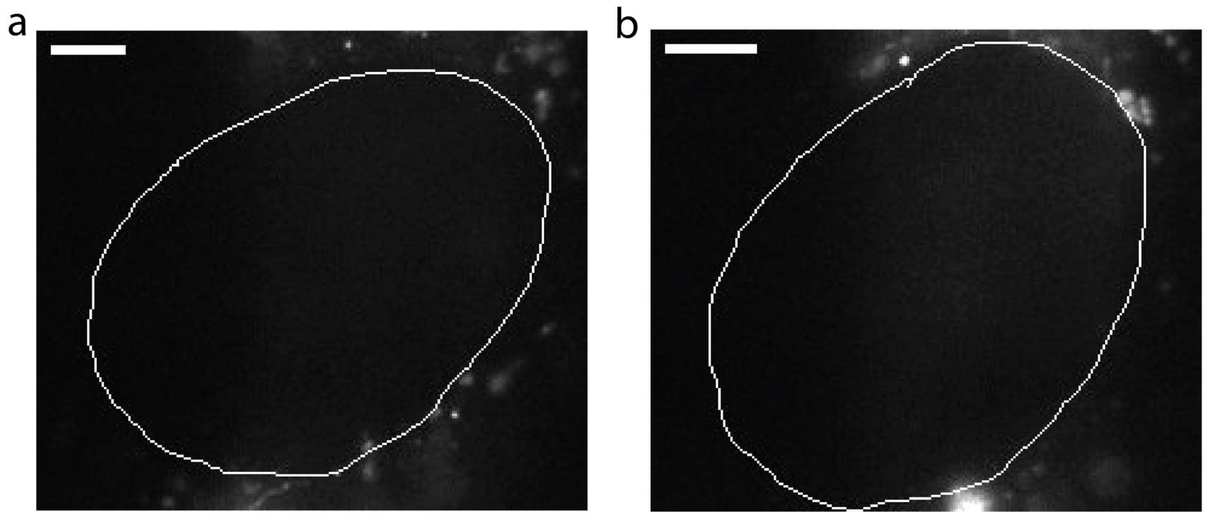

Supplementary Figure 4: Control of SiR-dye specificity. Standard deviations of 600 successive images (obtained with ImageJ) recorded for 30 s with 50 ms camera integration time of (**a**) a NIH3T3 cell and (**b**) the same cell as in (a) stained with 3 pM of SiR-Halo-tag dye according to the Halo-tag protocol (Promega). Scale bars are 5 µm. No SiR-Halo-dye signal is observed in the cell nucleus in the absence of a Halo-tag fusion protein.

Simulation (Figure)	Regularization Parameter	Bleaching rate (s^{-1})	Decay rates (s^{-1})	Time-lapse conditions (s)	Events per time-lapse
2a and 2b	0.01	1	0.1\5	0.05\0.13\0.32\0.80\2.00	1000
2c 1st line	0.01	1	0.011	0.0501\0.2\0.7079\2.66\10	100000
2c 2nd line	0.01	1	0.0107\7.0548	0.0501\0.2\0.7079\2.66\10	100000
2c 3rd line	0.01	1	0.0107\7.0548\0.0351	0.0501\0.2\0.7079\2.66\10	100000
2c 4th line	0.01	1	0.0107\7.0548\0.0351\0.1322	0.0501\0.2\0.7079\2.66\10	100000
2c 5th line	0.01	1	0.0107\7.0548\0.0351\0.1322\0.4977	0.0501\0.2\0.7079\2.66\10	100000
2c 6th line	0.01	1	0.0107\7.0548\0.0351\0.1322\0.4977\1.8738	0.0501\0.2\0.7079\2.66\10	100000
2d 1st line	0.01	1	0.0100\5	0.0501\0.0755\0.1136\0.1711\0.2577\0.3881\0.5843\0.8799\1.3250\1.9953	10000
2d 2nd line	0.01	1	0.0331\5	0.0501\0.0755\0.1136\0.1711\0.2577\0.3881\0.5843\0.8799\1.3250\1.9953	10000
2d 3rd line	0.01	1	0.1096\5	0.0501\0.0755\0.1136\0.1711\0.2577\0.3881\0.5843\0.8799\1.3250\1.9953	10000
2d 4th line	0.01	1	0.3631\5	0.0501\0.0755\0.1136\0.1711\0.2577\0.3881\0.5843\0.8799\1.3250\1.9953	10000
2d 5th line	0.01	1	1.2023\5	0.0501\0.0755\0.1136\0.1711\0.2577\0.3881\0.5843\0.8799\1.3250\1.9953	10000
2d 6th line	0.01	1	3.9811\5	0.0501\0.0755\0.1136\0.1711\0.2577\0.3881\0.5843\0.8799\1.3250\1.9953	10000
2e 1st line	0.01	20	0.0065\0.025\0.08\0.51\5.1	0.05\1\5\9	20000
2e 2nd line	0.01	7	0.0065\0.025\0.08\0.51\5.1	0.05\1\5\9	20000
2e 3rd line	0.01	2.4	0.0065\0.025\0.08\0.51\5.1	0.05\1\5\9	20000
2e 4th line	0.01	0.28	0.0065\0.025\0.08\0.51\5.1	0.05\1\5\9	20000
2e 5th line	0.01	0.1	0.0065\0.025\0.08\0.51\5.1	0.05\1\5\9	20000
2f 1st line	0.01	1	0.0065\0.025\0.08\0.51\5.1	0.05\1\5\9	10000

2f<br>2nd line	0.01	1	0.0065\0.025\0.08\0.51\5.1	0.05\1\5\9	10000
2f<br>3rd line	0.01	1	0.0065\0.025\0.08\0.51\5.1	0.05\1\5\9	10000
2f<br>4th line	0.01	1	0.0065\0.025\0.08\0.51\5.1	0.05\1\5\9	10000
2f<br>5th line	0.01	1	0.0065\0.025\0.08\0.51\5.1	0.05\1\5\9	10000
2g<br>1st line	0.01	1	0.0065\0.025\0.08\0.51\5.1	0.05\1\5\9	10000
2g<br>2nd line	0.01	1	0.0065\0.025\0.08\0.51\5.1	0.05\1\5\9	10000
2g<br>3rd line	0.01	1	0.0065\0.025\0.08\0.51\5.1	0.05\1\5\9	10000
2g<br>4th line	0.01	1	0.0065\0.025\0.08\0.51\5.1	0.05\1\5\9	10000
2g<br>5th line	0.01	1	0.0065\0.025\0.08\0.51\5.1	0.05\1\5\9	10000
2g<br>1st line	0	1	0.0160\0.2982\3.9194	0.05\0.1\2\10	250000
2g<br>2nd line	0	1	0.0142\0.0160\0.0180\0.2653\0.2982\0.3353\3.4864\3.9194\4.4062\	0.05\0.1\2\10	250000
2g<br>3rd line	0	1	0.0126\0.0142\0.0160\0.0180\0.0202\0.2360\0.2653\0.2982\0.3353\0.3769\3.1012\3.4864\3.9194\4.4062\4.9535	0.05\0.1\2\10	250000
2g<br>4th line	0	1	0.0112\0.0126\0.0142\0.0160\0.0180\0.0202\0.0227\0.2099\0.2360\0.2653\0.2982\0.3353\0.3769\0.4238\2.7585\3.1012\3.4864\3.9194\4.4062\4.9535\5.5688	0.05\0.1\2\10	250000
2g<br>5th line	0	1	0.0100\0.0112\0.0126\0.0142\0.0160\0.0180\0.0202\0.0227\0.0255\0.1867\0.2099\0.2360\0.2653\0.2982\0.3353\0.3769\0.4238\0.4764\2.4538\2.7585\	0.05\0.1\2\10	250000

			3.1012\3.4864\3.9194\4.4062\4.9535\5.5688\6.2605		
2h 1st line	0	1	k0=0.5\α=1	0.0501\0.1026\0.2100\0.4299\0.8799\1.8012\3.6869\7.5471\15.4486\31.6228	100000
2h 2nd line	0	1	k0=0.5\α=1.25	0.0501\0.1026\0.2100\0.4299\0.8799\1.8012\3.6869\7.5471\15.4486\31.6228	100000
2h 3rd line	0	1	k0=0.5\α=1.5	0.0501\0.1026\0.2100\0.4299\0.8799\1.8012\3.6869\7.5471\15.4486\31.6228	100000
2h 4th line	0	1	k0=0.5\α=1.75	0.0501\0.1026\0.2100\0.4299\0.8799\1.8012\3.6869\7.5471\15.4486\31.6228	100000
2h 5th line	0	1	k0=0.5\α=2	0.0501\0.1026\0.2100\0.4299\0.8799\1.8012\3.6869\7.5471\15.4486\31.6228	100000

Supplementary Table 1: Parameters used for the simulations in Figure 2 of the main text. The regularization parameter is inserted in GRID. The values for α indicate powerlaw exponents.

Cluster No.	1	2	3	4	5
Dissociation rate interval (1/s)	0.004-<br>0.007	0.018-<br>0.026	0.075-<br>0.090	0.50-<br>0.55	4.6-<br>5.2
All time-lapse conditions (Fig. 3b and Fig. 3d)					
event spectrum weight	(0.5 ± 0.2) %	(2.5 ± 0.6) %	(2.9 ± 0.7) %	(14.5 ± 1.3) %	(79.8 ±1.5) %
state spectrum weight	(30.3 ±7.4) %	(38.5 ± 5.5) %	(14.7 ± 3.6) %	(10.5 ± 1) %	(6.1 ± 0.6) %
without 0.05 s time-lapse condition (Supplementary Fig. 2b)					
event spectrum weight	(4.1 ± 0.6) %	(18.8 ± 1.4) %	(13.8 ± 1.3) %	(62.8 ± 1.4) %	n.d.
without 9 s time-lapse condition (Supplementary Fig. 2d)					
event spectrum weight	(0.2 ± 0.2) %	(1.6 ± 0.8) %	(4.2 ± 0.9) %	(13.5 ± 1.7) %	(80.5 ± 1.8) %

Supplementary Table 2: Positions and amplitudes of CDX2 dissociation rate clusters. Row 2 specifies the manually assigned dissociation rate intervals corresponding to a dissociation rate cluster. The spectral weight of each of the five distinct clusters of the CDX2-spectrum was obtained by integrating the GRID amplitudes resulting from 100% of the measured survival times. In line with Figure 3 b and d the spectral weights for the event spectrum and the state spectrum are shown separately. Errors of weights denote s.d. obtained from the 499 resampling runs.
